# Liquid crystal-amplified optofluidic biosensor for ultra-highly sensitive and stable protein assay

**DOI:** 10.1186/s43074-021-00041-1

**Published:** 2021-08-28

**Authors:** Ziyihui Wang, Yize Liu, Chaoyang Gong, Zhiyi Yuan, Liang Shen, Pengxiang Chang, Kun Liu, Tianhua Xu, Junfeng Jiang, Yu-Cheng Chen, Tiegen Liu

**Affiliations:** 1 School of Precision Instrument and Opto-Electronics Engineering, Tianjin University, Tianjin, 300072 China; 2 School of Electrical and Electronics Engineering, Nanyang Technological University, Singapore, 639798 Singapore; 3 School of Engineering, University of Warwick, Coventry, CV4 7AL UK

**Keywords:** Liquid crystal, Biosensor, Protein assay, Whispering-gallery mode, Microfluidic

## Abstract

Protein assays show great importance in medical research and disease diagnoses. Liquid crystals (LCs), as a branch of sensitive materials, offer promising applicability in the field of biosensing. Herein, we developed an ultrasensitive biosensor for the detection of low-concentration protein molecules, employing LC-amplified optofluidic resonators. In this design, the orientation of LCs was disturbed by immobilized protein molecules through the reduction of the vertical anchoring force from the alignment layer. A biosensing platform based on the whispering-gallery mode (WGM) from the LC-amplified optofluidic resonator was developed and explored, in which the spectral wavelength shift was monitored as the sensing parameter. The microbubble structure provided a stable and reliable WGM resonator with a high Q factor for LCs. It is demonstrated that the wall thickness of the microbubble played a key role in enhancing the sensitivity of the LC-amplified WGM microcavity. It is also found that protein molecules coated on the internal surface of microbubble led to their interactions with laser beams and the orientation transition of LCs. Both effects amplified the target information and triggered a sensitive wavelength shift in WGM spectra. A detection limit of 1 fM for bovine serum albumin (BSA) was achieved to demonstrate the high-sensitivity of our sensing platform in protein assays. Compared to the detection using a conventional polarized optical microscope (POM), the sensitivity was improved by seven orders of magnitude. Furthermore, multiple types of proteins and specific biosensing were also investigated to verify the potential of LC-amplified optofluidic resonators in the biomolecular detection. Our studies indicate that LC-amplified optofluidic resonators offer a new solution for the ultrasensitive real-time biosensing and the characterization of biomolecular interactions.

**Supplementary Information:**

The online version contains supplementary material available at 10.1186/s43074-021-00041-1.

## Introduction

Proteins, as essential components of organisms, play important roles in controlling biological activities and metabolism. Highly sensitive and selective identification and quantification of proteins are crucial due to their great importance for medical research and disease diagnoses in indicating pathological biological conditions [1, 2]. For example, albumin in human urine has a significant value in the diagnoses of diabetic nephropathy, cardiovascular and renal disease [3]. During current pandemic period, IgG and its antibody provide reliable biomarkers related to COVID-19 [4, 5], their detection can help prevent and control the virus infection at an early stage. However, routine technologies including mass spectrometry, enzyme-linked immunosorbent assays (ELISA) and fluorescence immunoassay, have varied capabilities for protein assay and there still remain several limitations due to high-cost and complex operations of such approaches [2]. Therefore, the development of a low-cost, stable and promising alternative method for protein detection is urgent and essential.

Liquid crystals (LCs), as an emerging branch of quickly-responsive, highly-sensitive, and low-cost materials, have been exploited in a wide variety of biosensing applications, especially for protein molecules detection [6–12]. LCs are employed to be signal amplifiers to report the subtle molecular events and external stimuli at LC interfaces. Over the past decade, the biological targets and sensing platforms of LC-based biosensors achieved rapid growth and became much diverse. Based on the orientation transition of LC molecules, the most widely used transduction method of LC-based biosensors is the monitoring under a polarized optical microscope (POM) [13, 14]. However, difficulties in distinguishing minor changes of LCs patterns via the naked eye constrain the sensitivity of POM approaches and their development in LC-based biosensors. Subsequently, various detection methods were also investigated. Some studies employed the image processing technique (for example, gray values of POM images) [15], dielectric properties of LC molecules [16, 17] and UV-vis/reflection spectra [18, 19] to analyze the variations in LC-based biosensors and to replace the naked eye observation. However, sensitivities of these reported works still need to be significantly improved. Recent advances have presented a method with a better sensitivity to detect the configuration of LCs based on whispering-gallery mode (WGM) laser emission spectra. This can offer a high signal intensity and a narrow laser linewidth to suppress the interference from noise. Taking the advantages of WGM lasering, LC microdroplets [20–22] and microfibers [23] were studied as sensing probes to realize the biomolecular detection. Nevertheless, such structures will be seriously affected by the external environment. For example, with the evaporation of the LC immersed liquid medium, the ordering of LC molecules will vary, which stops LC biosensors for long-term or multiple measurements. In addition, the free-space excitation reduced Q-factors of WGM cavities [24].

In this paper, with the merits of high Q factor, strong evanescent field and small mode volume from passive WGM cavities, a highly sensitive scheme for detecting protein molecules is proposed employing LC-amplified optofluidic resonators. The dimethyloctadecyl[3-(trimethoxysilyl)propyl] ammoniumchloride (DMOAP)-coated microcavity (with a shape of microbubble) can provide a WGM ring resonator with high stability and reliability, after the injection of LCs. Optofluidic resonators with thin walls can produce stronger light-matter interactions (in proximity to the capillary inner surface) and more flexible configurations. It is also observed that the vertical anchoring force from DMOAP is suppressed and LC molecules exhibit configuration transitions as the protein molecules (coated on the internal surface of the microcavity) increase. These will improve the sensitivity of resonance wavelength shifts, and can provide more parameter adjustment methods for optical sensing. With the triple effects of the biomolecular polarization, the orientation transition of LC molecules and the WGM, small variations in LC molecules can be amplified and caught, leading to the sensitive wavelength shift in spectra. Our results exhibited that the signal responses are enhanced with the increase of interferences from biomolecules, and a femtomole-level BSA at 1.92 fM (1.28 × 10^− 13^ g/ml) can be detected. Compared to conventional POM, the sensitivity of LC-amplified WGM biosensor was significantly improved, by seven orders of magnitude. Transferrin and Human IgG were also tested to verify the ability of our proposed sensor in protein assays. Moreover, specific biosensing for Human IgG and Anti-human IgG was demonstrated. Our scheme provides a multi-functional solution for label-free and real-time protein detection, with a significantly lower detection limit than that of conventional methods. Moreover, based on the wide applications of LCs in biosensing field, in addition to protein assays, such a promising technology can also be extended to sensitively detect more biomolecules in the future, by modifying the decoration of the microfluid surface.

## Materials and methods

### Materials and chemicals

For the fabrication of microcavity, the silica capillary was purchased from Polymicro Inc. (#TSP250350). Dimethyloctadecyl[3-(trimethoxysilyl)propyl] ammoniumchloride (DMOAP, #A-FF032) to modify the internal surface of the microcavity and 4-Cyano-4′-N-Pentylbiphenyl as nematic LC (5CB) were from Xianding Biotechnology Co., Ltd. (Shanghai, China, #C486A). When the wavelength is ranging from 1565 nm to 1575 nm, the extraordinary refractive index (*n*_*e*_) of 5CB is 1.67 and the ordinary refractive index (*n*_*o*_) of 5CB is 1.51. BSA (#PC0001from Solarbio Science & Technology, Co., Ltd., Beijing, China), transferrin (Solarbio, #T8010), human IgG (HIgG) (#bs-0297P from Bioss, Beijing, China), goat anti-human IgG (AHIgG) (Solarbio, #PA101), goat anti-human albumin (AHSA) (Solarbio, #SPA107) and goat anti-mouse IgG (AMIgG) (Solarbio, #SPA131) were used for protein assays as protein solutions. Olive oil (Solarbio, #O8320) was employed to explain the effects of absorption behavior on spectral responses.

### Preparation of microcavity and fiber taper

The fabrication of the microbubble (microcavity) has been reported in our previous work [25] and is separated into two steps. Firstly, the silica capillary is stretched via heating under the oxyhydrogen flame with a diameter of centimeter-scale. Then, the capillary is expanded and becomes a microbubble due to surface tension by switching into a smaller hydrogen oxide flame with a millimeter-level diameter and keeping the pressure constant. The entire process is carried out under a real-time monitoring device. Note that during the swelling process, the outer diameter and wall thickness can be controlled by adjusting the stretching length, the size of flame and the internal pressure. Using this method, microbubble resonators with the same diameter (210 μm) and various wall thicknesses were obtained. The Q factor of the microbubble cavity with a wall thickness of 3 μm is 7.034 × 10 [4]. These microcavities were fixed on the copper sheet by UV glue for protection. In addition, a fiber taper with a diameter of 1 ~ 2 μm was fabricated by the flame-heated stretching technique to excite the WGM.

### Functionalization of DMOAP and protein molecules

To form the alignment layer at the silica surface, an aqueous solution containing 0.01% (v/v) of DMOAP was injected into the microcavities for 30 mins at a flow rate of 0.7 μL/min, using a syringe pump (#SPLab01 from Shenchen Precision Pump Co., Ltd., Baoding, China). For the removal of excessive DMOAP molecules, deionized water was used to clean the surface for 1 min. Then, for the immobilization of biomolecules, protein solutions with various concentrations were filled into the DMOAP-coated microcavities for 30 mins, with the same pump setup, respectively, and unbound protein molecules were removed in the following step. Finally, LCs were injected into the DMOAP-coated microcavities for measurements of protein molecules.

### Optical setup

Polarized images were captured by a microscope (Coic DSZ2000X) with two cross polarizers and a charge-coupled device camera (Obvious U3CCD). For the WGM sensing system, the input laser beam was generated from a tunable laser (Keysight 81607A, linewidth < 10 kHz), ranging from 1565 nm to 1575 nm. Then the light beam was coupled into the LC-amplified optofluidic cavity through a fiber taper to acquire the WGM spectrum which was further received by the spectrometer (Yokogawa AQ6370).

## Results

### Effect of protein concentration variations on LC-amplified optofluidic resonator

**Fig. 1:**
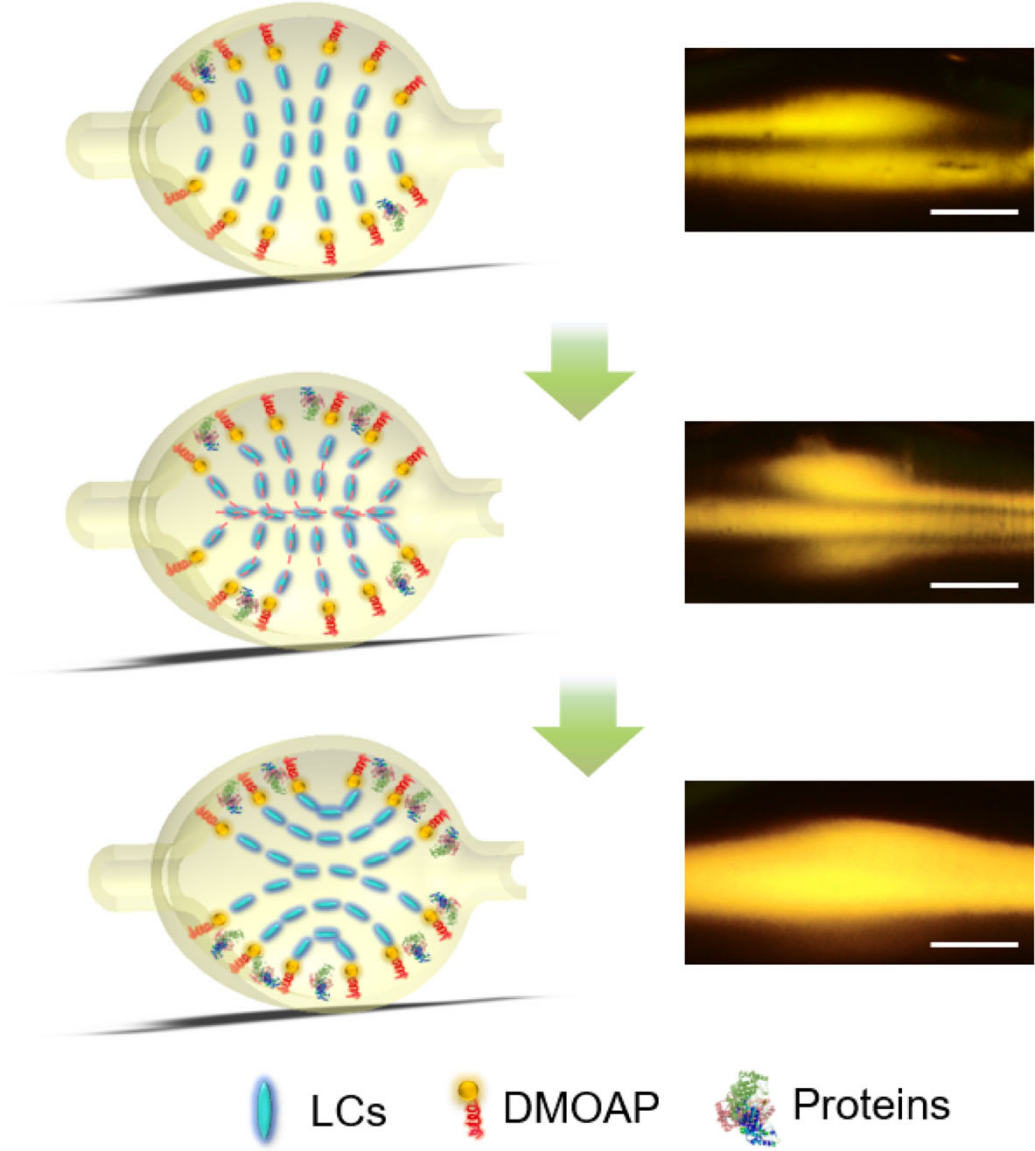
Under the disturbance of BSA molecules, the ordering of LC in microcavity is changed. With the increase of BSA concentrations, the illustrations of the ordering of LC molecules gradually changes towards the planer molecular orientation from top to bottom (left). The right panel shows the corresponding polarized optical images of LC-amplified optofluidic cavity. Scale bar: 100 μm

Firstly, we investigated the influence of protein molecules on the LC-amplified optofluidic cavity. The microcavity has a shape of microbubble, as seen in Fig. S1. The internal surface of the microcavity was coated with DMOAP throughout the entire study, which forces LC molecules to orient homeotropically to the surface of the microcavity. As shown in Fig. 1, with the addition of protein molecules, the vertical anchoring force from DMOAP will be suppressed by biomolecules [26], leading to the change of the orientation of LC molecules. Here we took BSA (protein standard) as an example and recorded the orientation of LCs filled in the microcavity via POM, where LCs were exposed to BSA, with various concentrations, for 15 mins, as shown in Fig. S2. Based on this operation, POM images at high concentrations of BSA (especially for 10^− 3^ g/ml, 10^− 4^ g/ml, and 10^− 5^ g/ml) show that the self-assembled configurations of LCs were changed and the BSA molecules caused the re-assembly of LC molecules. Such results indicate that for various concentrations of protein, LC-amplified optofluidic resonator can be used in the detection of biomolecules. However, for extremely low concentrations of biomolecules (below 10^− 6^ g/ml), small changes in LCs are very hard to be distinguished by the naked eye through POM and a more effective detection method needs to be developed.

### WGM spectra of LC-amplified optofluidic resonator

**Fig. 2:**
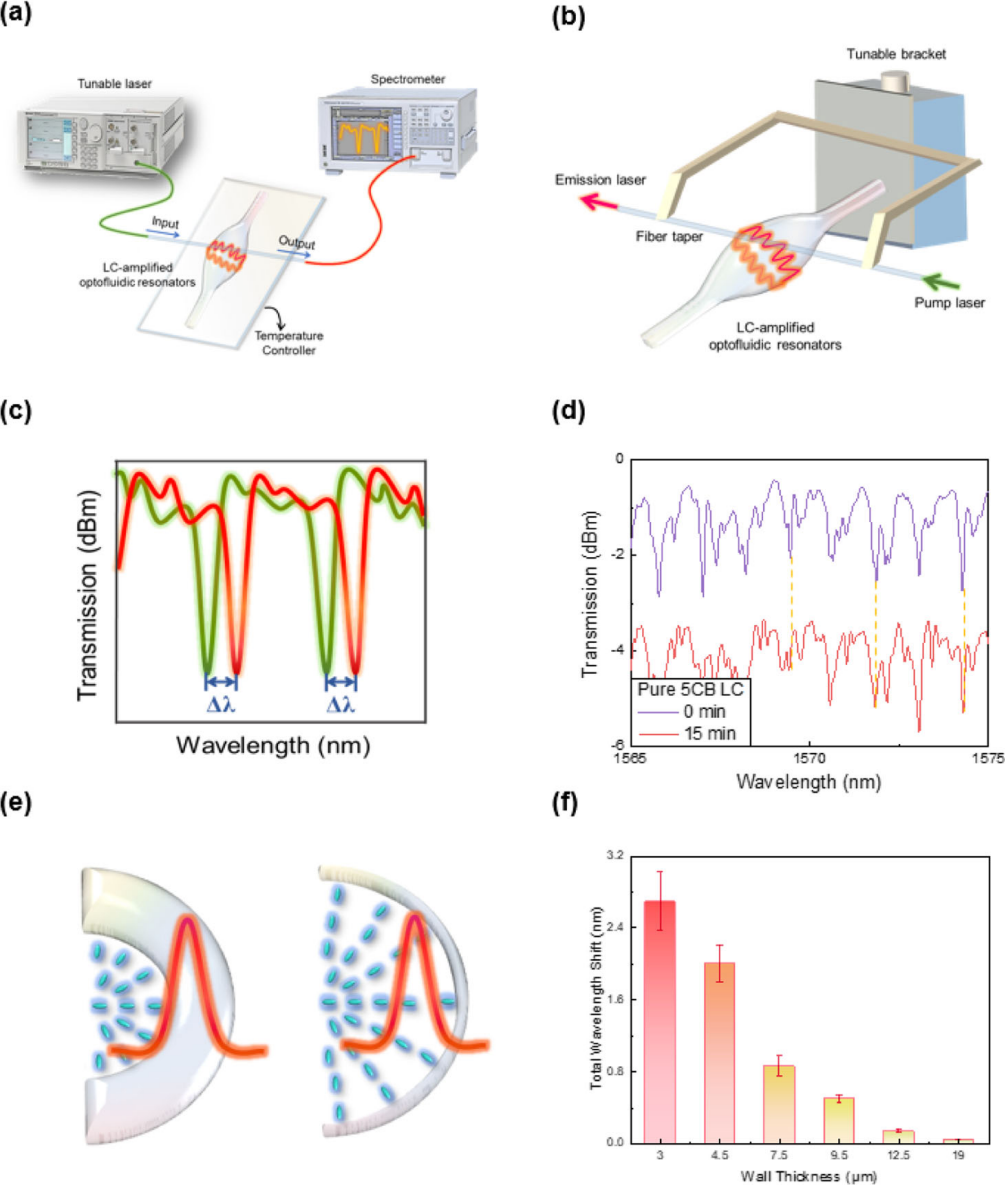
Schematic of the (**a**) experimental platform and (**b**) fiber taper-coupled micro-resonator (coupling position is controlled by tunable bracket). The light from a tunable laser was coupled into the LC-amplified optofluidic resonator through the fiber taper to acquire the WGM spectrum and was further received in spectrometer. The addition of protein molecules led to the orientation transition of LC and (**c**) the wavelength shift of WGM spectrum. (**d**) WGM laser spectral responses of LC-amplified optofluidic resonator under a controlled experiment (0 g/ml BSA) at 0 min (purple curve) and 15 mins (red curve), respectively. In addition, effect of the wall thickness on spectral responses was studied. (**e**) Illustration of intensity distribution of the incident laser beam for thick (left) and thin (right) wall of the microcavity. (**f**) WGM spectral responses of LC-amplified optofluidic resonator with various wall thicknesses at 1 mg/ml BSA within 15 mins

Based on the structure of LC-amplified optofluidic resonator, we developed a WGM resonator scheme to replace the conventional POM observation method, which provided a highly sensitive and cost-effective detection for protein molecules. To study the feasibility of the real-time monitoring of WGM spectra in the detection of protein molecules using LC-amplified optofluidic resonator (the micro-resonator produced by silica can provide a stable WGM cavity for LCs and eliminate the impact from external environment), a continuously tunable laser from 1565 nm to 1575 nm was used as the pump source and coupled to the micro-resonator via the fiber taper, as shown in Fig. 2a. The fiber taper has merits of ultra-high coupling efficiency (up to 99%) [27], easy-fabricating and convenient for integration. To realize an efficient coupling, the adjustment of phase matching and the overlap of evanescent electromagnetic fields are critical and can be controlled by the tunable bracket, as depicted in Fig. 2b. The tunable bracket can change the gap between fiber taper and the microcavity. 4-Cyano-4′-N-Pentylbiphenyl (5CB), as a thermotropic LC, was employed in this study. In this sensing system, the optofluidic cavity was placed on a temperature controller to avoid external factors affecting the precision of the biomolecule detection. External stimuli from protein molecules lead to the orientation transition of LC molecules, as such, the corresponding resonant spectrum will shift and the WGM signals are received by a spectrometer for further processing, as seen in Fig. 2c. In addition, the sharp transmission dips exhibit the excitation of WGMs inside the amplified optofluidic resonator, and valley wavelengths correspond to optical modes.

According to the previous study [26], a lower flow speed for filling LCs into the microchannel can increase the response-ability of LCs on biomolecule detection. In our study, LCs were filled into the microcavity by a pump with a low flow speed of 0.7 μL/min to ensure an efficient intermolecular reaction. In addition, when the injection of LCs is finished, UV glue was used to seal both ends of the optofluidic cavity to keep the balance of the internal air pressure and to reduce the flow speed of LCs. We started to record the changes of WGM spectra after the arrival of LCs at the optofluidic microresonator by 15 s. This can also reduce the influence of LC fluidity on the signal responses. As a controlled experiment, Fig. 2d showed that the stability of WGM spectra from LC-amplified optofluidic cavity (with DMOAP and 0 g/ml BSA modified on the surface), and only a minor spectral wavelength shift can be detected (<< 0.1 nm). It indicates that our method provides a stable and reliable structure and can be used for further biological sensing. In addition, according to the WGM spectra, the Q factor of the LC-amplified optofluidic cavity was calculated to be 1.4 × 10^4^ by *Q* = *λ/δλ* (where *λ* is the valley wavelength and *δλ* is the linewidth). Compared to WGM in other reported LC microcavities (Q factors of LC microdroplets and LC microfibers: ~ 10^3^) [23, 28, 29], the Q factor of our sensing platform was considerable increased, which indicates that LC-amplified optofluidic cavity biosensors offer great potential in high-sensitivity detection of protein.

A tunable bracket was employed to change the position of the fiber taper and to ensure the overlap of evanescent fields. With the coupling between the fiber and the optofluidic cavity, the photon (migration path) will firstly experience the silica. As seen in Fig. 2e and Fig. S3, if the wall of the silica microcavity is sufficiently thin, the evanescent field will be coupled into the interior of the optofluidic cavity and fed into LCs for monitoring the orientation transition of LC molecules. Otherwise, light will only travel along the silica region and the variation of LCs cannot be detected. To understand the key factors in affecting the sensitivity of biological detection, the impact of wall thickness on the spectral wavelength shift was studied under the same condition. As a demonstration, 1 mg/ml BSA was coated onto the surface of the optofluidic cavity to disturb the vertical anchoring force from the alignment layer (DMOAP). For various wall thicknesses (3 μm, 4.5 μm, 7.5 μm, 9.5 μm, 12.5 μm and 19 μm), corresponding wavelength responses were presented in Fig. 2f (The calculation of the wall thickness is described in the section of Support Information). It is found that the wall thickness has a significant effect on the detection sensitivity. LC molecules at the surface can communicate their orientations within a vicinity of up to 100 μm [30]. The smaller the wall thickness is, the stronger the light intensity can be fed into the LC region. Accordingly, the orientation transition of LC molecules can be more comprehensively monitored via the transmission spectral shift. Therefore, the sensitivity of the molecular detection can be improved by controlling the wall thickness of the microcavity. Hereafter, the LC-amplified optofluidic cavity biosensor with a wall thickness of 3 μm was used in all following studies.

In addition, the WGM mode can be predicted using the following equation [29, 31, 32]:

$$ {\uplambda}^{-1}\left(\mathrm{R},{\mathrm{n}}_1,{\mathrm{n}}_{\mathrm{r}},\mathrm{q},\mathrm{m}\right)=\frac{1}{2\uppi \mathrm{R}{\mathrm{n}}_1}\left[\mathrm{m}+\frac{1}{2}+{2}^{-\frac{1}{3}}\upalpha \left(\mathrm{q}\right){\left(\mathrm{m}+\frac{1}{2}\right)}^{\frac{1}{3}}-\frac{\mathrm{L}}{{\left({\mathrm{n}}_{\mathrm{r}}^2-1\right)}^{\frac{1}{2}}}+\frac{3}{10}{2}^{-\frac{2}{3}}{\upalpha}^2\left(\mathrm{q}\right){\left(\mathrm{m}+\frac{1}{2}\right)}^{-\frac{1}{3}}-{2}^{-\frac{1}{3}}\mathrm{L}\left({\mathrm{n}}_{\mathrm{r}}^2-\frac{2}{3}{\mathrm{L}}^2\right)\frac{\upalpha \left(\mathrm{q}\right){\left(\mathrm{m}+\frac{1}{2}\right)}^{-\frac{2}{3}}}{{\left({\mathrm{n}}_{\mathrm{r}}^2-1\right)}^{\frac{2}{3}}}\right], $$

where R is the radius of the optofluidic cavity, n_1_ represents the refractive index of the surrounding medium, n_r_ = n_1_/n_2_ (n_2_ is the refractive index of the microcavity), α(q) and m are the roots of the Airy function (q exhibits the mode order) and the mode number, respectively. For the TM mode, L = 1/n_r_, and for the TE mode, L = n_r_. According to the result in Fig. S3, we set n_1_ = 1.44 (silica) and n_2_ = 1.67 (*n*_*e*_). The spectrum can be well-fitted with first-order TM polarization modes (q = 1) from 648 to 651, and second-order TM polarization modes (q = 2) from 635 to 638, as seen in Fig. S4.

### WGM spectral responses of LC-amplified optofluidic resonator induced by protein molecules

**Fig. 3:**
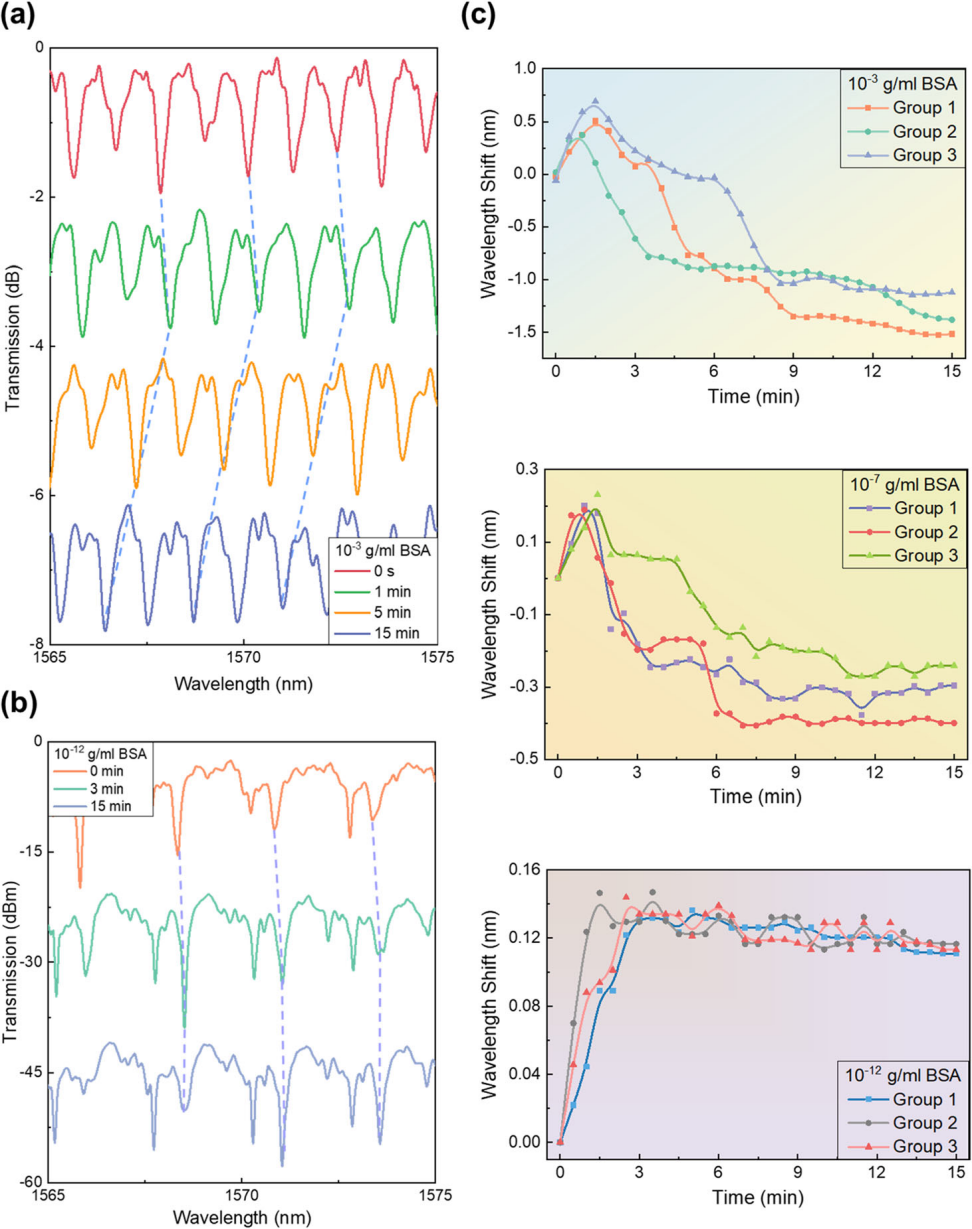
WGM laser spectra of the LC-amplified optofluidic resonator under (**a**) 10^− 3^ g/ml BSA (at 0, 1, 5, 15 mins, respectively) and (**b**) 10^− 12^ g/ml BSA (at 0 and 15 mins). (**c**) Real-time monitoring of WGM laser spectral shift of the LC-amplified optofluidic resonator under different BSA concentrations (10^− 3^, 10^− 7^, 10^− 12^ g/ml BSA, from top to bottom) within 15 mins. (Points in the same group are connected by B-spline curve)

In this section, the potential of the LC-amplified optofluidic cavity for biosensing was explored. The alignment layer on the internal surface of the silica microcavity, formed by DMOAP, can be disturbed by protein molecules, and the light-matter interactions will also occur at the internal surface of micro-resonator. Hence this sensing platform exhibits a great applicability on the detection of various protein molecules. At first, we tested BSA as a target molecule at different concentrations. To suppress the external effects, e.g. the flow speed of LCs, on spectra, we started to continuously record the variation of WGM spectra, 15 s after the stop of the LC injection. Figure 3a showed wavelength shift responses of transmission spectra from the LC-amplified optofluidic cavity for the 10^− 3^ g/ml BSA, corresponding to the POM image in Fig. 1c with 10^− 3^ g/ml BSA, where an obvious orientation change of LC molecules can also be observed. By contrast, for the 10^− 12^ g/ml BSA, the minor changes in POM images were difficult to be distinguished via the naked eye observation. However, the transmission spectra showed clear wavelength shift responses within the same duration. This indicates that WGM spectra were more sensitive than conventional polarized images on the detection of protein molecules and can provide better capability for biomolecules sensing. Moreover, we also monitored the traces of spectral responses at three typical concentrations of BSA (10^− 3^ g/ml, 10^− 7^ g/ml and 10^− 12^ g/ml), as seen from top to bottom in Fig. 3c. For each concentration of BSA, three groups of data were recorded and the three groups exhibited similar tendency. Interestingly, under the various concentrations of BSA, spectral responses showed red-shift at first and then displayed blue-shift tendency.

**Fig. 4:**
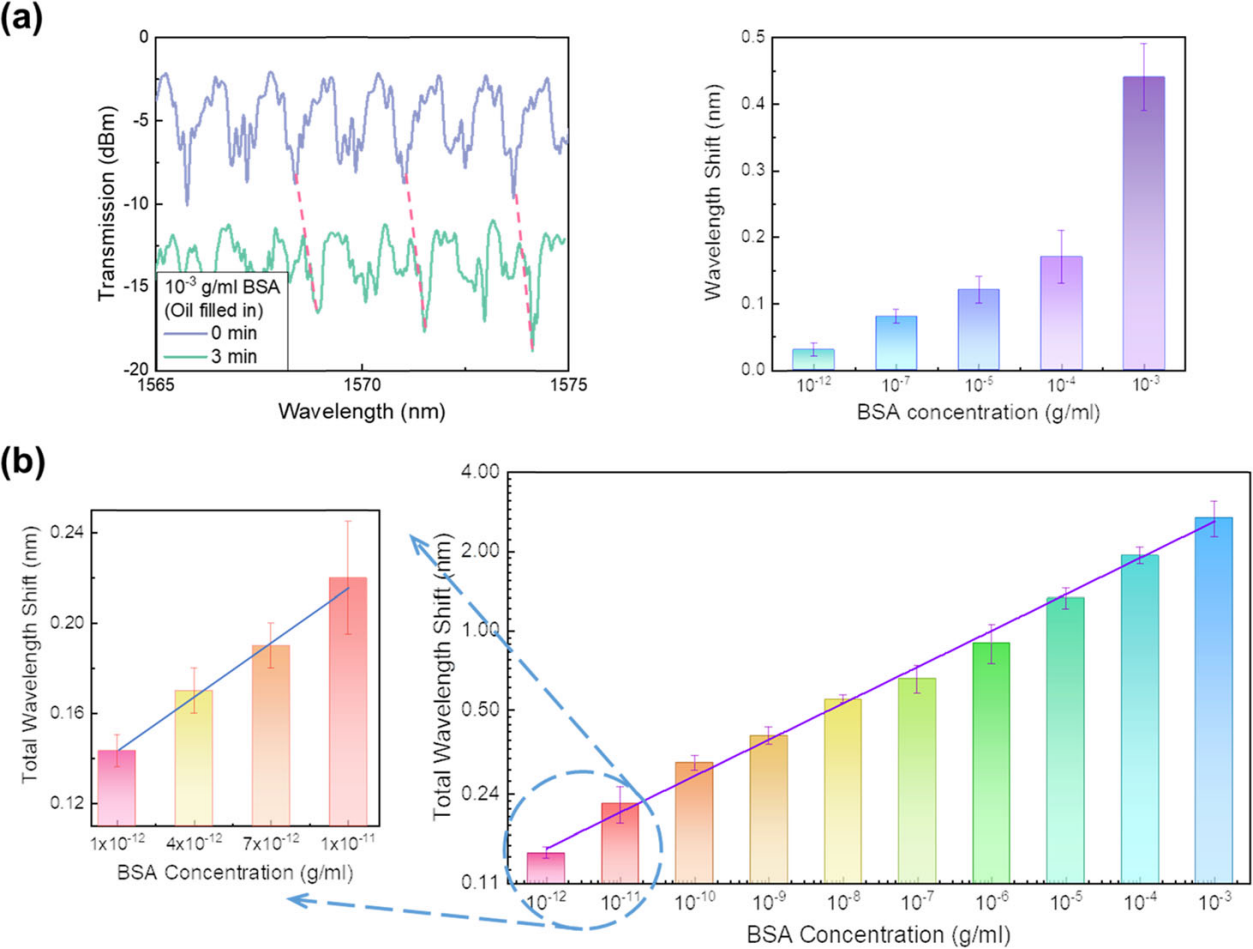
(**a**) Oil was filled in resonator to study the effect of BSA absorption behavior on WGM spectral wavelength shift. The WGM spectral responses at 10^− 3^ g/ml BSA (left panel) showed only red-shift tendency and the overall wavelength shift under several BSA concentrations was shown in right panel. (**b**) The overall wavelength shift responses from WGM laser spectra under various BSA concentrations (from 10^− 12^ to 10^− 3^ g/ml) are calculated by summing up the blue shift distance and the red shift distance. The left panel exhibits the corresponding wavelength responses at various extremely low BSA concentrations (10^− 12^, 4 × 10^− 12^, 7 × 10^− 12^ and 10^− 11^ g/ml). According to the calibration curve, the limit of detection for BSA is 1.92 fM (1.28 × 10^− 13^ g/ml)

For the radial orientation of LCs without any BSA molecules, as seen in Fig. 2e, the TM mode with the oscillating electric field along the long axis of the LC molecules experienced the extraordinary refractive index (*n*_*e*_) [31]. With the orientation transition of LC molecules (due to the addition of BSA molecules), the TM mode responds to the ordinary refractive index (*n*_*o*_) of LCs. The wavelength shifts with respect to the changes in the refractive index [33, 34] (from *n*_*e*_ to *n*_*o*_) showed a gradually blue-shift tendency. However, it is also noted that WGM spectra showed a red-shift at first, which behaved opposite to the subsequant blue-shift tendency according to the orientation transition of LC molecules. In fact, the resonance frequency is sensitive to the change in the close vicinity of the cavity surface [35, 36]. Herein, we suppose the occurrence of the red-shift was arisen from the BSA absorption behavior at the LC-silica interface. With the absorption of BSA, the interactions between the evanescent field of the WGM and the BSA will polarize the molecules, leading to the perturbation in the energy of a single-photon resonant state and wavelength shift [36–38]. To support this viewpoint, oil was used to replace LCs for responding to BSA solutions, as oil would not have the effect of orientation transition during the absorption of BSA. In this experiment, the DMOAP-coated optofluidic cavity sensor was modified by BSA with several concentrations and was then filled with oil at a speed of 0.7 μL/min. In accordance with Fig. 3c, wavelength responses did not show significant red-shift tendency after three mins. Therefore, for demonstration, WGM spectra from the oil-filled optofluidic resonator were monitored within three mins. At first, when the sensor surface was modified with 0 g/ml BSA, there was no significant wavelength shift (data not shown) in the WGM spectra from the oil-filled optofluidic resonator. In Fig. 4a, with the decoration of BSA, only a red-shift tendency was detected in spectra (left panel) and the responses had a positive correlation with the concentrations of BSA (right panel). It is demonstrated that the absorption behavior of BSA could trigger the red shift of WGM spectra. Therefore, the wavelength responses in transmission spectra arised from the joint effect of the adsorption of BSA at the LC-aqueous interface and the orientation transition of LCs (due to the BSA interference and changes in alignment layers). At first, the adsorption resulted in stronger spectral responses which concealed the small blue-shift tendency from the orientation transition of LCs. Afterward, the orientation transition of LC molecules became dominant and led to the blue-shift trend. Both factors were amplified by WGM spectra and triggered the wavelength shift.

Regardless of the direction of shift, for a higher concentration of BSA, the shift effect was always stronger because higher-density BSA molecules participated in the interactions with laser, which led to the orientation transition of LCs. According to Fig. 3c, the wavelength shift approached its saturation, meaning the equilibrium of LCs in the resonator, around 15 mins after the reactions between LCs and BSA. Therefore, we calculated the overall wavelength shift (absolute value of red-shift + absolute value of blue-shift) within 15 mins (more detailed descriptions regarding the total wavelength shift can be found in Fig. S5). For the same concentration of BSA, the overall wavelength responses were similar. According to the above results, WGM spectral shifts under various concentrations of BSA, from 10^− 12^ g/ml to 10^− 3^ g/ml, were tested respectively, each for 15 mins. Results of overall signal responses against corresponding BSA concentrations were plotted, with a logarithmic scale, in Fig. 4b. The maximum signal responses at 10^− 3^ g/ml BSA reached 2.69 nm and the minimum spectral shift at 10^− 12^ g/ml BSA was about 0.14 nm. Several extremely low concentrations of BSA between 10^− 12^ g/ml and 10^− 11^ g/ml were also exhibited (Fig. 4b). The overall wavelength shifts of spectral responses depend linearly on the BSA concentrations and a calibration curve of these results was provided. Based on the curve-fitting, the limit of detection (LOD) was calculated, which was 1.92 fM (1.28 × 10^− 13^ g/ml) BSA by 3σ/k (the standard deviation σ = 0.000342; the slope of the calibration curve k = 8.048 × 10^− 9^). According to the POM images shown in Fig. 1c, only when the concentration of BSA exceeded 10^− 6^ g/ml, the variation of LC molecules could be distinguished. The minor orientation transition of LC molecules, which cannot be observed by the naked eye, can now be easily caught via WGM spectra. Compared to reported works (listed in Table 1), the LOD of our LC-amplified microfluidic resonator is two orders of magnitude better than the lowest LOD achieved in reported LC-based sensing systems. This indicates that our LC-amplified optofluidic resonator sensing platform has the capability of ultra-sensitive (femtomole-level) protein assays.

**Table 1:** Comparison on LOD of LC based sensing systems in detecting BSA

Detection methods	Limit of Detection	References
Transmission spectra of DLC cells	10^−6^ g/ml	[39]
Electric field tuned DFLC cell	10^− 7^ g/ml	[8]
Electric capacitance of LC cells	10^−9^ g/ml	[17]
Transmission spectra of BPLC cells	10^−9^ g/ml	[40]
WGM ring resonator (without LCs amplified)	2 × 10^− 10^ g/ml	[41]
WGM lasing spectra of LC droplet	2.4 × 10^−11^ g/ml	[20]
LC-amplified WGM microfluidic resonator	1.28 × 10^− 13^ g/ml (1.92 fM)	This study

**Fig. 5:**
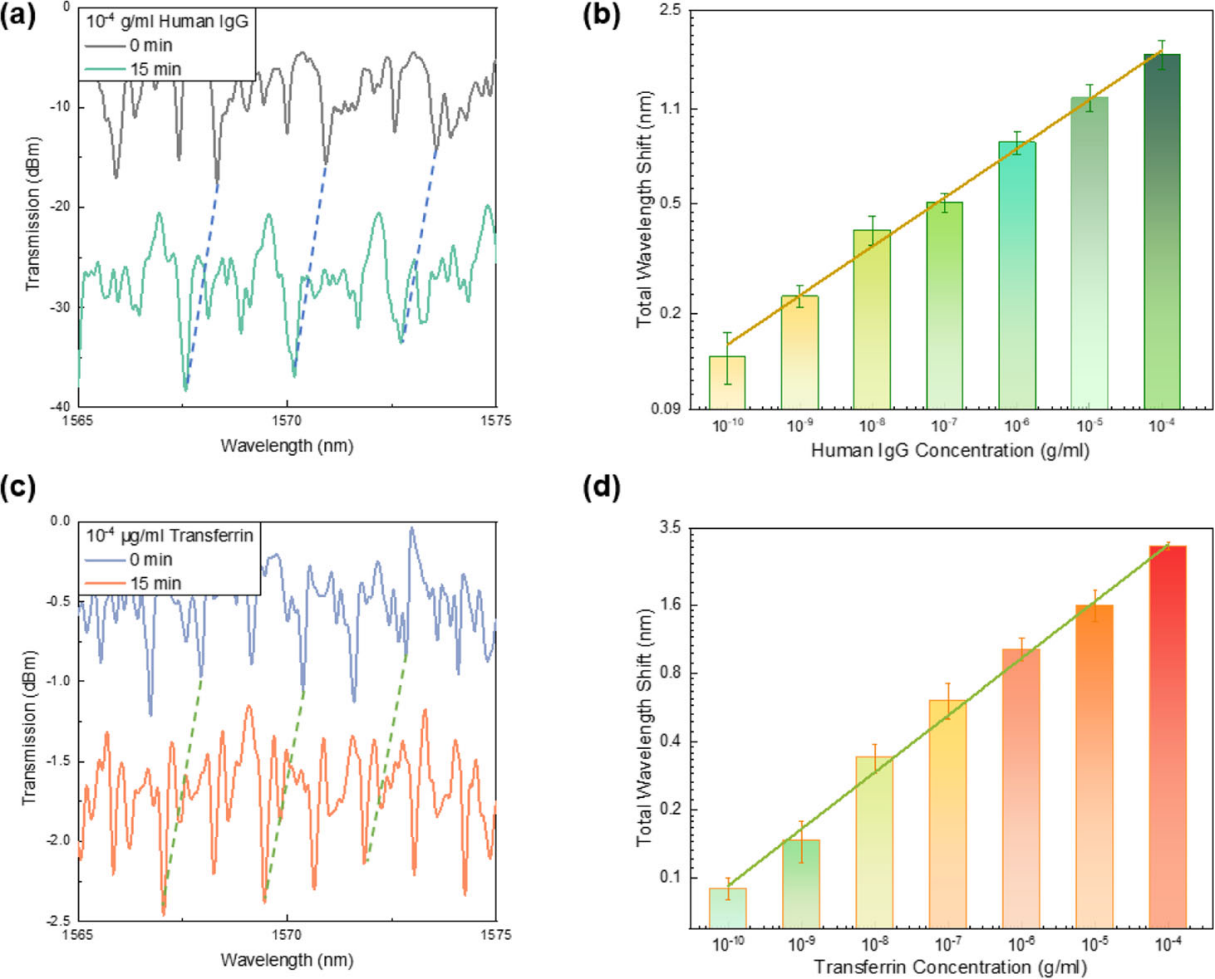
WGM laser spectra of the LC-amplified optofluidic resonator under 10^− 4^ g/ml (**a**) human IgG and (**c**) transferrin at 0 min and 15 mins. The overall wavelength shift responses under different concentrations of (**b**) human IgG and (**d**) transferrin

To demonstrate the universality of the LC-amplified optofluidic resonator on protein assays, various concentrations of human IgG (HIgG) and transferrin were also tested in this paper. The signal responses on the detection of 10^− 4^ g/ml HIgG were shown in Fig. 5a. The wavelength shifts of transmission spectra in the HIgG sensing showed a similar trend as those in BSA sensing (red-shift at first and then blue-shift). The overall responses versus corresponding HIgG concentrations were plotted in Fig. 5b, where a linear relationship between HIgG concentrations and spectral shifts was also observed. Moreover, Fig. 5c decribes the spectral shift for the test of 10^− 4^ g/ml transferrin and the WGM responses also scaled linearly with the concentrations of transferrin, as shown in Fig. 5d. According to the above results, our biosensing platform worked well for these proteins and showed a good potential for ultra-sensitive detection on various protein molecules.

### Potential application for specific biosensing

**Fig. 6:**
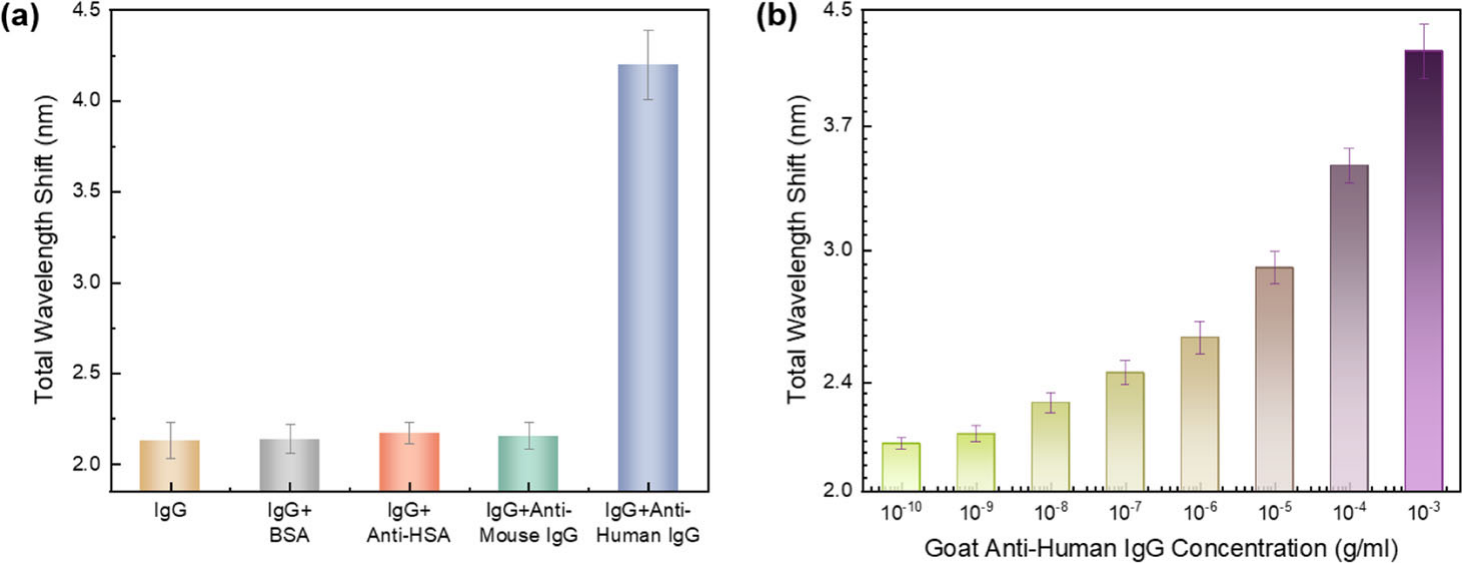
Effects of specific binding between anti-human IgG and human IgG. (**a**) With 10^− 3^ g/ml human IgG coated on the silica wall, WGM wavelength shift responses when LCs are exposed to various proteins (BSA, anti-HSA, anti-mouse IgG and anti-human IgG) at the same concentration of 10^− 3^ g/ml. (**b**) WGM spectral shift resulted from antibody-antigen binding events between 10^− 3^ g/ml Human IgG and different concentrations of goat anti-human IgG

In addition, to verify the feasibility of specific biosensing, we carried out an experiment for detecting specific binding events, using HIgG and anti-human IgG (AHIgG). Herein, 1 mg/ml HIgG (throughout this section) was used as a substrate to modify the inner surface of the DMOAP-coated microcavity for 12 h at 4 °C (to prevent the inactivation of the protein), and 1 mg/ml BSA was used to check whether the unbound sites were left on the microcavity inner surface. At this point, after the injection of LCs, the wavelength shift responses of the HIgG + BSA modified surface were monitored and were compared to the responses of HIgG (only)-modified surface, as shown in Fig. 6a. There was no significant difference in the overall shift (around 2.13 nm) between these two groups. This means that the bound sites of micro-resonator surface were almost fully occupied by HIgG. In the following, HIgG-modified microcavity was exposed to anti-HAS (AHSA), anti-mouse IgG (AMIgG) and AHIgG, respectively, and then excessive biomolecules were removed. These analyte solutions were all at a concentration of 1 mg/ml. As shown in Fig. 6a, compared to HIgG or HIgG + BSA, when the sensor was immersed in AHSA and AMIgG, transmission spectra did not display obvious change in responses. However, after the addition of AHIgG, transmission spectral shift reached 4.2 nm due to the immunobinding between AHIgG and HIgG. Therefore, various concentrations of AHIgG were characterized via spectral wavelength shift and were plotted, with the logarithmic scale, in Fig. 6b. Because HIgG was employed as the substrate, the spectral responses for the detection of analyte molecules were larger than 2.13 nm (HIgG added only). The overall wavelength shifts showed a positive correlation with the AHIgG concentrations. This indicates that our sensing platform possesses high selectivity owing to the underlying immune reactions. As a result, the proposed LC-amplified optofluidic resonator biosensor can be used for the specific detection of various proteins with high sensitivity, especially for biomarkers, e.g. carcinoembryonic antigen (CEA, cancer markers), detection in disease diagnoses.

## Discussion

In this study, we exploited a highly sensitive LC-amplified optofluidic resonator biosensor based on WGM spectra for the quantitative analysis of proteins. To support WGM, the amplified optofluidic resonator was employed to provide a stable and reliable ring resonator for LCs. With the assistance of the external tunable laser and the fiber taper, interrogations of the WGM and enhancements in the Q factor of the micro-resonator were achieved. It is found that the wall thickness of the LC-amplified optofluidic WGM micro-resonator has a major impact on the sensitivity of sensing since the light intensity can be better distributed in the core of the resonator. The protein immobilized on the internal surface of the DMOAP-coated microcavity triggered the orientation transition of LC molecules. Moreover, due to the interaction between biomolecules and the laser beam, RI of protein molecules was changed accordingly. Under the joint effects from these two factors, WGM spectra can amplify such subtle variation and characterize the concentration of target molecules via wavelength shift. BSA was taken as the first example for the protein molecule, the overall spectral shift increased linearly with the BSA concentrations, and a limit of detection at a femtomole level (1.92 fM, namely 1.28 × 10^− 13^ g/ml BSA) was achieved. Compared to the conventional POM observation, the sensitivity of such a WGM-based biosensor was improved by seven orders of magnitude. Other protein molecules (transferrin and human IgG) and specific binding experiments using human IgG and anti-human IgG were also investigated to show the applicability of the proposed sensing platform for protein assays. With the improvement of the manufacturing process and the Q factor of the microcavity, our sensing platform can provide a better limit of detection for protein assay. In addition, the spectral analysis can be handled by real-time image processing approaches, e.g., correlation operation etc., to replace manual computations. Therefore, a more efficient detection method based on the LC-amplified optofluidic biosensor can be realized. Owing to the wide applications of LCs in biosensing field, besides protein assay, this developed LC sensing platform can offer promising solutions for the label-free biomolecular detections with high sensitivity by modifying the decoration of microfluidic cavities to further trigger the orientation transition of LCs.

## Supplementary Information

Additional file 1: **Figure S1.** Bright-field of LC-amplified microcavity with a shape of microbubble. Scale bar: 100 μm. **Figure S2.** Polarized optical images of LC-amplified optofluidic cavity under various BSA concentrations (from 10^− 12^ g/ml to 10^− 3^ g/ml, and 0 g/ml as a control group). Scale bar: 100 μm. **Figure S3.** Electromagnetic profiles of optical modes supported by the LC-amplified microfluidic resonator with a thin (left, 3 μm) and thick (right, 19 μm) wall. In each illustration, media from left to right refer to LCs, silica and air, respectively. For the cavity with a thin wall, the light intensity is mainly focused in the LC region (due to the high refraction index of LC molecules), and first-order and second-order polarization modes are formed. On the contrary, rays will only travel along the silica wall and the orientation transition of LC molecules cannot be monitored, which leads to a low-sensitivity. Scale bar: 25 μm. **Figure S4.** The WGM resonance observed in the LC-amplified microfluidic cavity, which corresponds to the first-order (mode number *m* from 648 to 651) and the second-order (mode number *m* from 635 to 638) TM polarization modes. **Figure S5.** Illustrative example of the total wavelength shift in the orientation transition of LC molecules. The total wavelength shift equals the sum of the absolute value of the red-shift and the absolute value of the blue-shift: (1) The absolute value of the red-shift equals the Y-axis coordinate of point A (i.e., the top point of the curve),which also refers to the distance of the red line; (2) The absolute value of blue-shift equals the difference at Y-axis coordinate between point A and point B (i.e., the peak-to-peak difference of the curve or the distance of the blue line when the X-axis coordinate is 15). **Figure S6.** Illustration of the shape of the microcapillary and the microbubble. The heating length is *L*_0_, and the elongation length is *x*. (a) The original microcapillary. The initial outer and inner radii are *r*_*out*0_ and *r*_*in*0_, respectively. (b) The stretched microcapillary. The outer and inner radii of the tapered waist are *r*_*out*1_ and *r*_*in*1_, respectively. (c) The microbubble fabricated from the microcapillary in (b). The outer radius is *r*_*out*2_ and the inter radius is *r*_*in*2_. **Figure S7.** Sectional view of the microbubble. (a) Normal microbubble. (b) Microbubble used in this work.

## Data Availability

The original data are available on request from corresponding authors.
