# Supplementary material for: Liquid crystal-amplified optofluidic biosensor for ultra-highly sensitive and stable protein assay
Source: Photonix. 2021 Aug 28;2(1):18. doi: 10.1186/s43074-021-00041-1 (PMC8397869; doi:10.1186/s43074-021-00041-1)
Supplement: Supplementary file 1 — Figure S1. Bright-field of LC-amplified microcavity with a shape of microbubble. Scale bar: 100 μm. Figure S2. Polarized optical images of LC-amplified optofluidic cavity under various BSA concentrations (from 10− 12 g/ml to 10− 3 g/ml, and 0 g/ml as a control group). Scale bar: 100 μm. Figure S3. Electromagnetic profiles of optical modes supported by the LC-amplified microfluidic resonator with a thin (left, 3 μm) and thick (right, 19 μm) wall. In each illustration, media from left to right refer to LCs, silica and air, respectively. For the cavity with a thin wall, the light intensity is mainly focused in the LC region (due to the high refraction index of LC molecules), and first-order and second-order polarization modes are formed. On the contrary, rays will only travel along the silica wall and the orientation transition of LC molecules cannot be monitored, which leads to a low-sensitivity. Scale bar: 25 μm. Figure S4. The WGM resonance observed in the LC-amplified microfluidic cavity, which corresponds to the first-order (mode number m from 648 to 651) and the second-order (mode number m from 635 to 638) TM polarization modes. Figure S5. Illustrative example of the total wavelength shift in the orientation transition of LC molecules. The total wavelength shift equals the sum of the absolute value of the red-shift and the absolute value of the blue-shift: (1) The absolute value of the red-shift equals the Y-axis coordinate of point A (i.e., the top point of the curve),which also refers to the distance of the red line; (2) The absolute value of blue-shift equals the difference at Y-axis coordinate between point A and point B (i.e., the peak-to-peak difference of the curve or the distance of the blue line when the X-axis coordinate is 15). Figure S6. Illustration of the shape of the microcapillary and the microbubble. The heating length is L0, and the elongation length is x. (a) The original microcapillary. The initial outer and inner radii are rout0 and rin0, res [file 43074_2021_41_MOESM1_ESM.docx]

**Support Information**

**
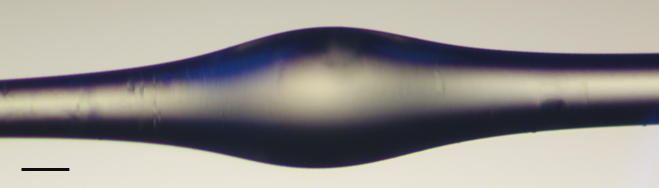
**

**Fig. S1** Bright-field of LC-amplified microcavity with a shape of microbubble. Scale bar: 100 μm.

**
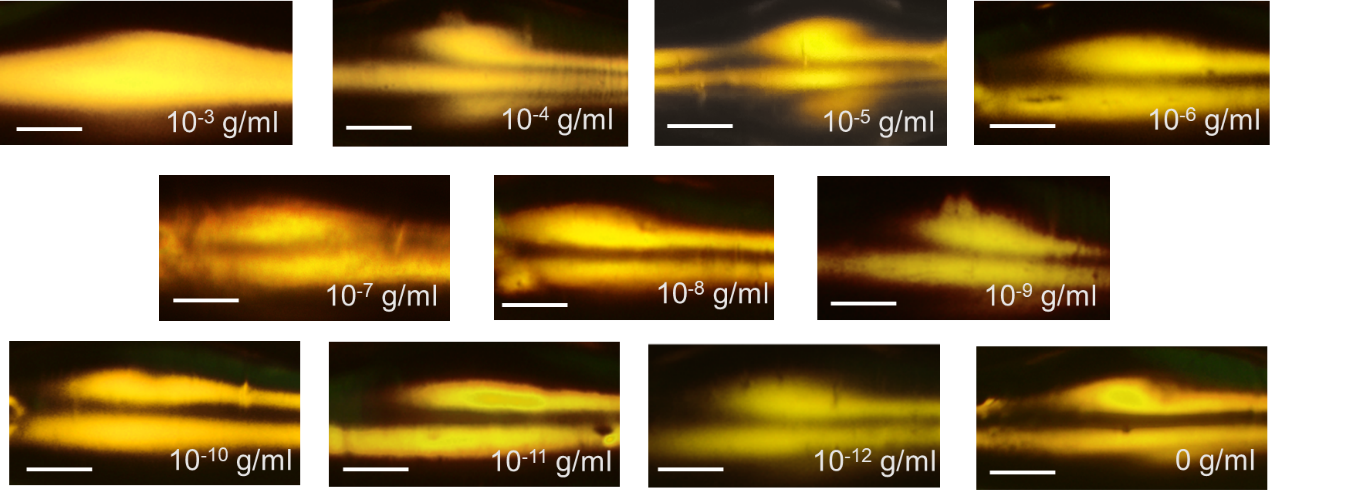
**

**Fig. S2** Polarized optical images of LC-amplified optofluidic cavity under various BSA concentrations (from 10^-12^ g/ml to 10^-3^ g/ml, and 0 g/ml as a control group). Scale bar: 100 μm.


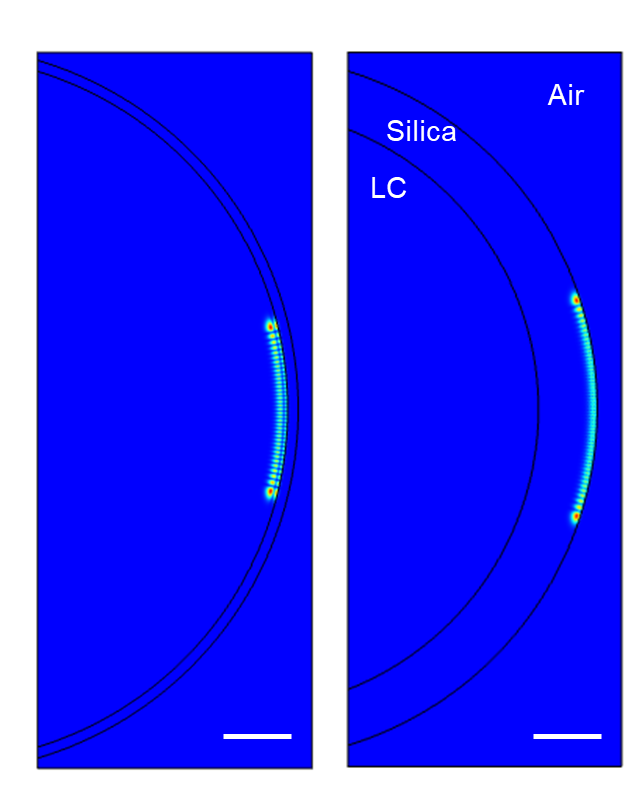


**Fig. S3** Electromagnetic profiles of optical modes supported by the LC-amplified microfluidic resonator with a thin (left, 3 μm) and thick (right, 19 μm) wall. In each illustration, media from left to right refer to LCs, silica and air, respectively. For the cavity with a thin wall, the light intensity is mainly focused in the LC region (due to the high refraction index of LC molecules), and first-order and second-order polarization modes are formed. On the contrary, rays will only travel along the silica wall and the orientation transition of LC molecules cannot be monitored, which leads to a low-sensitivity. Scale bar: 25μm.


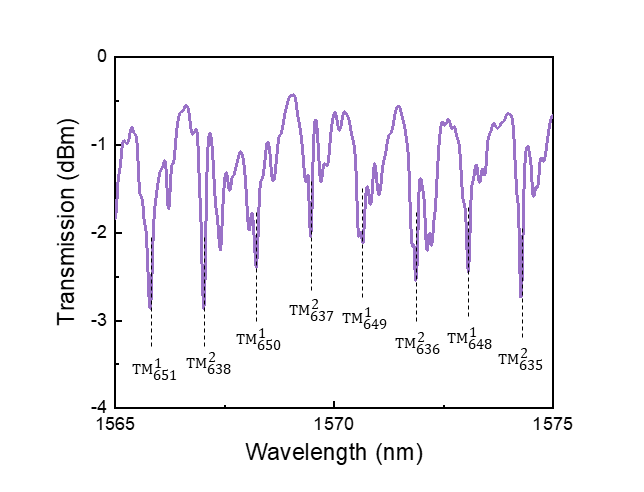


**Fig. S4** The WGM resonance observed in the LC-amplified microfluidic cavity, which corresponds to the first-order (mode number *m* from 648 to 651) and the second-order (mode number *m* from 635 to 638) TM polarization modes.


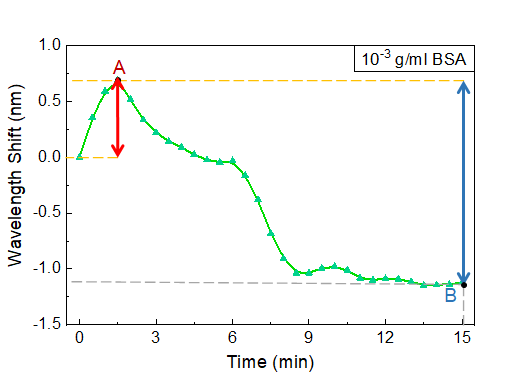


**Fig. S5** Illustrative example of the total wavelength shift in the orientation transition of LC molecules. The total wavelength shift equals the sum of the absolute value of the red-shift and the absolute value of the blue-shift: (1) The absolute value of the red-shift equals the Y-axis coordinate of point A (i.e., the top point of the curve),which also refers to the distance of the red line; (2) The absolute value of blue-shift equals the difference at Y-axis coordinate between point A and point B (i.e., the peak-to-peak difference of the curve or the distance of the blue line when the X-axis coordinate is 15).

**Calculation of the wall thickness**

In our previous work, a wall-thickness-controlled microbubble fabrication model for the WGM-based application was developed.^25^ According to the fabrication process, the wall thickness of the microfluidic resonator can be calculated using the following two steps:

Fig. S6 exhibited variations of the shape and the radii during the whole fabrication process.


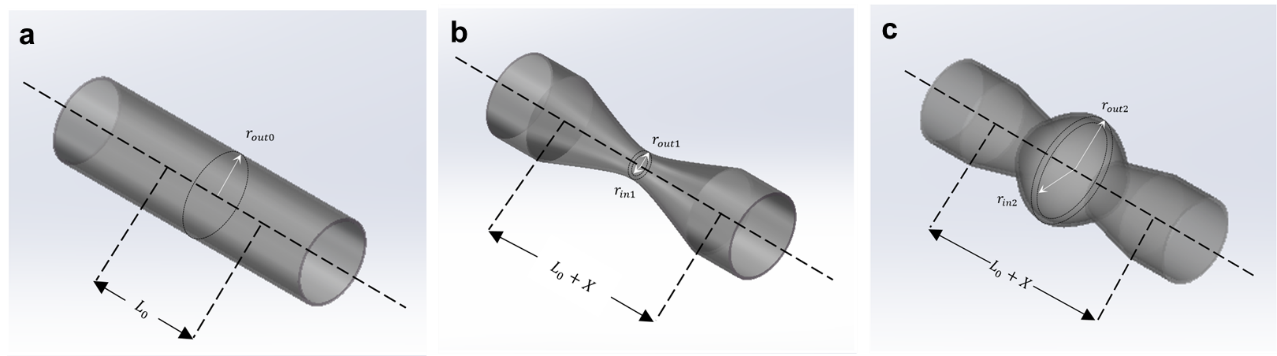


**Fig. S6** Illustration of the shape of the microcapillary and the microbubble. The heating length is $L_{0}$, and the elongation length is *x*. (a) The original microcapillary. The initial outer and inner radii are $r_{out0}$ and $r_{in0}$, respectively. (b) The stretched microcapillary. The outer and inner radii of the tapered waist are $r_{out1}$ and $r_{in1}$, respectively. (c) The microbubble fabricated from the microcapillary in (b). The outer radius is $r_{out2}$ and the inter radius is $r_{in2}$.

(1) Tapering. It considers the radius of the stretched microcapillary under the effect of the elongation. When the microcapillary is stretched during the heating process, the radius of the microcapillary can be expressed as:

$\frac{\boldsymbol{r}_{\boldsymbol{out}\boldsymbol{0}}}{\boldsymbol{r}_{\boldsymbol{in}\boldsymbol{0}}}\boldsymbol{=}\frac{\boldsymbol{r}_{\boldsymbol{out}\boldsymbol{1}}\boldsymbol{(x)}}{\boldsymbol{r}_{\boldsymbol{in}\boldsymbol{1}}\boldsymbol{(x)}}$ **(S1)**

where $r_{out0}$ and $r_{in0}$ are the outer and the inner radii of the original microcapillary, respectively, $r_{out1}(x)$ and $r_{in1}(x)$ are the outer and the inner radii after the stretching, which are also related to the elongation length *x*. $r_{out1}(x)$ can be determined via the advanced calibration experiment, and $r_{in1}(x)$ can be calculated accordingly.

(2) Swelling. In this step we consider the change of the wall thickness during the formation of the microbubble with the assistance of the pressure (controlled by the air volume in the syringe). Due to mass conservation, there is no change on the silica volume during the fabrication of the microcapillary waist. The structure parameters of the microcapillary waist keep constant along the axis direction. Due to the spherical symmetry of the micro-resonator, the volume of the microbubble and the corresponding microcapillary waist part, i.e., $V_{microbubble}$ and $V_{microcapillary}$, can be given by:

$$\boldsymbol{V}_{\boldsymbol{microcapillary}}\boldsymbol{=}\left( \boldsymbol{\pi}\boldsymbol{\cdot}\boldsymbol{r}_{\boldsymbol{out}1}^{\boldsymbol{2}}\boldsymbol{-}\boldsymbol{\pi}\boldsymbol{\cdot}\boldsymbol{r}_{\boldsymbol{in}1}^{\boldsymbol{2}} \right)\boldsymbol{\times}\boldsymbol{2}\boldsymbol{r}_{\boldsymbol{out}2}$$

$\boldsymbol{V}_{\boldsymbol{microbubble}}\boldsymbol{=}\left( \frac{\boldsymbol{4}}{\boldsymbol{3}}\boldsymbol{\pi}\boldsymbol{\cdot}\boldsymbol{r}_{\boldsymbol{out}2}^{\boldsymbol{3}}\boldsymbol{-}\frac{\boldsymbol{4}}{\boldsymbol{3}}\boldsymbol{\pi}\boldsymbol{\cdot}\boldsymbol{r}_{\boldsymbol{in}2}^{\boldsymbol{3}} \right)\boldsymbol{\times}\left( \frac{\boldsymbol{4}\boldsymbol{\pi}\boldsymbol{\cdot}\boldsymbol{r}_{\boldsymbol{out}2}^{\boldsymbol{2}}\boldsymbol{-}\boldsymbol{2}\boldsymbol{\pi}\boldsymbol{\cdot}\boldsymbol{r}_{\boldsymbol{out}1}^{\boldsymbol{2}}}{\boldsymbol{4}\boldsymbol{\pi}\boldsymbol{\cdot}\boldsymbol{r}_{\boldsymbol{out}2}^{\boldsymbol{2}}} \right)$ **(S2)**

where $r_{out2}$ and $r_{in2}$ are the outer and the inner radii of the microbubble, respectively. Based on the hollow structure of the microcapillary (with two open ends), circular areas (as shown in **Fig. S7**) on both sides of the microbubble, with radii of $r_{out1}$, need to be subtracted when calculating the shell volume.


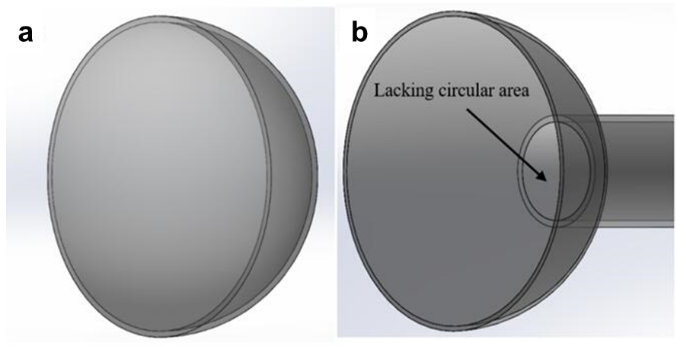


**Fig. S7** Sectional view of the microbubble. (a)Normal microbubble. (b) Microbubble used in this work.

Since $V_{microbubble}=V_{microcapillary}$(mass conservation), the wall thickness $t_{1}$ of the microbubble can be calculated as:

$\boldsymbol{t}_{\boldsymbol{1}}\boldsymbol{=}\boldsymbol{r}_{\boldsymbol{out}2}\boldsymbol{-}\boldsymbol{r}_{\boldsymbol{in}2}\boldsymbol{=}\boldsymbol{r}_{\boldsymbol{out}2}\boldsymbol{-}\left[ \boldsymbol{r}_{\boldsymbol{out}2}^{\boldsymbol{3}}\boldsymbol{-}\frac{\boldsymbol{6}\boldsymbol{r}_{\boldsymbol{out}2}\boldsymbol{\cdot}\left( \boldsymbol{r}_{\boldsymbol{out}1}^{\boldsymbol{2}}\boldsymbol{-}\boldsymbol{r}_{\boldsymbol{in}1}^{\boldsymbol{2}} \right)}{\boldsymbol{4}\left( \boldsymbol{1}\boldsymbol{-}\frac{\boldsymbol{r}_{\boldsymbol{out}1}^{\boldsymbol{2}}}{\boldsymbol{2}\boldsymbol{r}_{\boldsymbol{out}2}^{\boldsymbol{2}}} \right)} \right]^{\frac{\boldsymbol{1}}{\boldsymbol{3}}}$ **(S3)**
